# Nutritional Status and Cognition in Older Adults: Modification by Apolipoprotein E ε4

**DOI:** 10.3390/nu18193217

**Published:** 2026-09-29

**Authors:** Young Min Choe, Ji-Hyun Kim, Hye Ji Choi, Boung Chul Lee, Guk-Hee Suh, Shin Gyeom Kim, Hyun Soo Kim, Jaeuk Hwang, Dahyun Yi, Jee Wook Kim

**Affiliations:** 1 Department of Neuropsychiatry, Hallym University Dongtan Sacred Heart Hospital, 7 Keunjaebong-gil, Hwaseong 18450, Gyeonggi, Republic of Korea; ymchoe7@gmail.com (Y.M.C.); jihyun.kim@hallym.or.kr (J.-H.K.); phahyethon@hanmail.net (H.J.C.); gukheesuh@naver.com (G.-H.S.); 2 Department of Psychiatry, Hallym University College of Medicine, Chuncheon 24252, Gangwon, Republic of Korea; leeboungchul@hallym.or.kr; 3 Department of Neuropsychiatry, Hallym University Hangang Sacred Heart Hospital, Seoul 07247, Republic of Korea; 4 Department of Neuropsychiatry, Soonchunhyang University Bucheon Hospital, Bucheon 14584, Gyeonggi, Republic of Korea; redmensch@schmc.ac.kr; 5 Department of Laboratory Medicine, Hallym University Dongtan Sacred Heart Hospital, 7 Keunjaebong-gil, Hwaseong 18450, Gyeonggi, Republic of Korea; kimhyun@hallym.or.kr; 6 Department of Psychiatry, Soonchunhyang University Hospital Seoul, Seoul 04401, Republic of Korea; hju75@schmc.ac.kr; 7 Institute of Human Behavioral Medicine, Medical Research Center Seoul National University, Seoul 03080, Republic of Korea; dahyunyi@gmail.com

**Keywords:** nutritional status, Mini-Nutritional Assessment, cognition, apolipoprotein E ε4, protein intake, calf circumference, Alzheimer’s disease

## Abstract

**Background**: Malnutrition is a modifiable risk factor for late-life cognitive decline, but whether its cognitive correlates vary by genetic risk is unclear. We examined whether the apolipoprotein E ε4 allele (APOE4) modifies associations of the Mini-Nutritional Assessment (MNA) and its subscales with global cognition. **Methods**: In this cross-sectional analysis of 196 non-demented older Korean adults from the General Lifestyle and Alzheimer’s Disease (GLAD) cohort, multiple linear regression tested associations of MNA total (MNA-T), screening (MNA-S), and assessment (MNA-A) scores with the CERAD total score (TS), including MNA × APOE4 interactions. Exploratory analyses examined one representative item per a priori nutritional domain. **Results**: After adjustment, MNA-T (*p* = 0.006) and MNA-A (*p* = 0.003), but not MNA-S (*p* = 0.065), were associated with TS. APOE4 carrier status modified these associations (interaction *p* = 0.005 and 0.007, respectively); associations were evident in non-carriers but not in carriers, an underpowered subgroup (*n* = 40). Among the domain-representative items, only self-rated health survived Bonferroni correction (*p* = 0.006); protein intake (*p* = 0.025) and calf circumference (*p* = 0.043) showed nominal associations only. **Conclusions**: Better nutritional status was associated with better global cognition, predominantly in APOE4 non-carriers. These hypothesis-generating findings require confirmation in larger longitudinal and interventional studies.

## 1. Introduction

Age-related cognitive decline and Alzheimer’s disease (AD) impose a major global burden, and identifying modifiable factors that shape cognitive trajectories has become a public health priority [1,2]. Among such factors, nutritional status is particularly attractive because it is measurable in routine clinical care, dynamic across the lifespan, and amenable to intervention [3,4]. Older adults are uniquely susceptible to malnutrition owing to physiological changes in appetite regulation, sensory function, gastrointestinal motility, low-grade chronic inflammation, multimorbidity, and polypharmacy [3,5]. Across community-dwelling older populations, the prevalence of malnutrition or risk of malnutrition consistently exceeds 20–30% in vulnerable subgroups [6,7].

Cross-sectional and longitudinal studies have linked poor nutritional status with worse cognitive performance and greater risk of mild cognitive impairment (MCI) and dementia [8,9,10,11,12]. The Mini-Nutritional Assessment (MNA) is one of the most widely used and best-validated tools for evaluating nutritional status in older adults [13,14]. The 18-item MNA is composed of a brief screening subsection (MNA-S, items A–F, max 14 points) that captures recent changes in food intake, weight, mobility, acute disease, neuropsychological problems, and body mass index (BMI), and a more detailed assessment subsection (MNA-A, items G–R, max 16 points) covering living circumstances, medication use, pressure sores, meal frequency, dietary content (protein, fruit/vegetable, fluid), mode of feeding, self-perceived nutrition and health, mid-arm circumference (MAC), and calf circumference (CC). Lower MNA total score (MNA-T) is consistently associated with worse cognition and faster cognitive decline [9,10,11,12]. However, three important questions remain unresolved.

First, the screening and assessment subscales may convey non-redundant information about cognition, yet most prior studies report only MNA-T. Whether the brief screening subscale, which can be administered in minutes, captures the cognitively relevant signal—or whether the more detailed assessment subscale is required—has clear implications for routine clinical practice. Second, the apolipoprotein E ε4 allele (APOE4), the strongest common genetic risk factor for late-onset AD, shapes systemic metabolism, lipid handling, neuroinflammatory tone, and amyloid-β deposition [15,16,17,18]. Because nutrition acts on the brain in part through metabolic and inflammatory pathways that overlap with those modulated by APOE4, it is biologically plausible that the association between nutritional status and cognition is not uniform across genotypes. One hypothesis is that amyloidogenic and neuroinflammatory processes in APOE4 carriers might attenuate the observable association between nutritional status and cognition. Genotype-specific effects on cognition have been described for body composition, dietary patterns, and physical activity [19,20], and an umbrella review suggests that the cognitive effects of dietary interventions may differ by APOE4 carrier status [21]; however, few studies have formally tested APOE4 as a moderator of the association between MNA-defined nutritional status and cognition. Third, the MNA aggregates 18 heterogeneous items spanning anthropometry, dietary content, subjective health, and functional status, and the relative contribution of each component to cognition has rarely been examined. Consequently, it remains unclear whether the MNA–cognition association reflects a single underlying construct or, instead, independent contributions from conceptually distinct nutritional domains. Identifying which specific items are associated with cognition could help identify which components of nutritional status merit investigation as candidate targets.

In this study of non-demented older adults from the General Lifestyle and AD (GLAD) cohort, we addressed these three gaps. We deliberately focused on the non-demented stage, in which nutritional status is still potentially modifiable and a preventive window for cognitive preservation remains open, before dementia-related declines in appetite and food intake confound the relationship. We examined whether MNA-T, MNA-S, and MNA-A are each associated with global cognition, indexed by the Consortium to Establish a Registry for Alzheimer’s Disease (CERAD) total score (TS); we tested whether APOE4 status moderates these associations using formal interaction terms and stratified analyses, hypothesizing that the association between better nutritional status and cognition would be more pronounced—and possibly limited to—APOE4 non-carriers; and we grouped the 18 individual MNA items a priori into three nutritional domains and, using within-domain exploratory selection followed by a multivariable model including all three domain-representative items, identified the representative item of each domain, to characterize which specific components carry the cognitive signal. By integrating subscale-level, genotype-stratified, and item-level analyses within a single cohort, this work moves beyond the conventional MNA-T analysis toward a more granular and clinically relevant understanding of the nutrition–cognition relationship in late life.

## 2. Materials and Methods

### 2.1. Participants

The present study is a cross-sectional analysis of baseline data from the GLAD (General Lifestyle and AD) study, an ongoing prospective cohort launched in 2020. By July 2022, 196 dementia-free adults between 65 and 90 years of age had been enrolled; of these, 83 were classified as cognitively normal (CN) and 113 as having mild cognitive impairment (MCI). Enrollment proceeded via a memory-clinic dementia screening program at Hallym University Dongtan Sacred Heart Hospital (Hwaseong, Republic of Korea), with additional cases identified through community outreach. CN classification required a Clinical Dementia Rating (CDR) of 0 together with no prior MCI or dementia [22]. MCI diagnosis followed standard amnestic criteria—informant-confirmed memory concerns, measurable memory deficits, preserved overall cognitive function, independence in daily living activities, and no evidence of dementia. The memory-impairment threshold was defined as a demographically (age-, education-, and sex-) adjusted z-score of −1.0 or lower on at least one of the four episodic-memory subtests of the Korean CERAD battery—word list memory, recall, and recognition, plus constructional recall [23,24,25]. A CDR of 0.5 was required for all MCI cases. We excluded anyone with major psychiatric or neurological disease, comorbid conditions likely to compromise cognition, illiteracy, marked sensory loss, communication or behavioral barriers to reliable assessment, or current use of investigational drugs. Of the 227 individuals who volunteered for eligibility assessment, 31 were excluded: 9 met an exclusion criterion (major psychiatric disorder, *n* = 3; neurological or medical condition affecting cognition, *n* = 4; illiteracy, sensory impairment, or communication or behavioral problems, *n* = 1; use of investigational drugs, *n* = 1), 2 could not be classified as CN or MCI, and 20 withdrew consent (*n* = 17) or were lost to contact (*n* = 3). All 196 enrolled participants had complete data for all MNA items, covariates, biomarkers, and cognitive outcomes, and were included in the analyses; no imputation was required. Among the 40 APOE4 carriers, 39 were APOE ε3/ε4 heterozygotes and 1 was an ε4/ε4 homozygote.

### 2.2. Clinical Assessments

Each participant completed a thorough clinical workup performed by trained psychiatrists following the GLAD protocol, which incorporates the clinical and neuropsychological battery of CERAD [23,24]. The CERAD neuropsychological tests were administered by licensed psychologists experienced in working with older populations [25]. Final diagnoses were reached by consensus among psychiatrists and psychologists with expertise in dementia. The primary outcome, global cognition, was indexed by the CERAD total score (TS) [26]—a composite obtained by adding seven subtest scores (verbal fluency, modified Boston Naming Test, word list memory, constructional praxis, word list recall, word list recognition, and constructional recall), with higher scores reflecting better overall cognitive performance.

Vascular burden was ascertained through structured interviews with participants and their family informants. A vascular risk score (VRS), reported as a percentage, was generated by summing the presence of six conditions: hypertension, diabetes mellitus, dyslipidemia, coronary heart disease, transient ischemic attack, and stroke [27]. Participants were grouped into three economic tiers based on whether annual household income fell below, at, or above the minimum cost of living defined by Korea’s Ministry of Health and Welfare. Physical activity was quantified with the Korean version of the Physical Activity Scale for the Elderly (PASE) [28,29]. Lifetime drinking (never/former/current) and smoking (never/ex-smoker/current) histories were obtained from interviews supplemented by medical-record reviews.

### 2.3. Nutritional Assessment

Nutritional status was assessed with the full 18-item Mini-Nutritional Assessment (MNA), a tool validated for older populations [13,14]. Its total score (MNA-T, range 0–30) combines a screening component (MNA-S, range 0–14; items A–F) and an assessment component (MNA-A, range 0–16; items G–R). The screening items address (A) decline in food intake, (B) weight loss, (C) mobility, (D) psychological stress or acute disease, (E) neuropsychological problems, and (F) body mass index. MNA-A items include (G) living independently, (H) prescription medication count, (I) pressure sores, (J) number of full meals per day, (K) selected consumption markers for protein intake (dairy ≥ 1 serving/day; legumes or eggs ≥ 2 servings/week; meat, fish, or poultry daily), (L) fruit and vegetable intake, (M) fluid intake, (N) mode of feeding, (O) self-view of nutritional status, (P) self-rated health relative to people of the same age, (Q) mid-arm circumference, and (R) calf circumference [14]. Anthropometric items in the MNA assessment subsection—MAC and CC—were measured following standard MNA protocols, with each measurement taken twice and the average recorded to minimize measurement error. To ensure reliability, informants were interviewed when necessary [13].

### 2.4. Blood Biomarkers

Blood samples were collected in the morning (8–9 A.M.) via venipuncture using trace element-free tubes. Serum albumin, fasting glucose, and high-density lipoprotein (HDL)- and low-density lipoprotein (LDL)-cholesterol were quantified on the COBAS c702 platform with the manufacturer’s reagents (Roche Diagnostics, Mannheim, Germany) under centralized quality assurance.

### 2.5. APOE4 Genotyping

Genomic DNA was extracted from EDTA-anticoagulated whole blood with the QIAamp DSP DNA Blood Mini Kit (QIAGEN, Hilden, Germany). APOE alleles were typed using the Seeplex ApoE ACE kit (Seegene, Seoul, Republic of Korea) amplified on a ProFlex thermal cycler (ThermoFisher Scientific, Waltham, MA, USA), and amplicons were resolved on the QIAxcel Advanced System (QIAGEN, Hilden, Germany). The resulting genotypes (ε2/ε2, ε2/ε3, ε2/ε4, ε3/ε3, ε3/ε4, ε4/ε4) were dichotomized such that participants carrying at least one ε4 allele were classified as APOE4 carriers and all others as APOE4 non-carriers.

### 2.6. Statistical Analysis

The association between nutritional status (MNA-T, MNA-S, MNA-A) and global cognition (TS) was evaluated using multiple linear regression analyses. Each MNA score was modeled as a continuous independent variable, with TS as the dependent variable. Two predefined models were estimated: an unadjusted Model 1 and a fully adjusted Model 2 controlling for age, sex, APOE4 status, education, diagnostic group (CN vs. MCI), VRS, PASE score, annual income, lifetime alcohol and smoking histories, albumin, fasting glucose, and HDL- and LDL-cholesterol. Model 2 included 14 covariates, providing at least 10 participants per parameter in the full sample even when categorical covariates were counted as separate indicator variables; this exceeds the minimum of approximately two subjects per variable reported to be sufficient for accurate estimation of regression coefficients, standard errors, and confidence intervals in linear regression [30]. Within the APOE4 carrier stratum (*n* = 40), this ratio approached that minimum; inference about effect modification was therefore based on interaction terms estimated in the full sample, and the parsimonious core-adjusted model described below served as the principal check on the stratified estimates. To account for three primary MNA exposures tested against one outcome, a Bonferroni-corrected threshold of *p* < 0.0167 (0.05/3) was applied, with nominal *p* < 0.05 also reported.

To assess possible effect modification by APOE4, the models were extended with two-way interaction terms between each MNA score and APOE4 carrier status, and the cohort was additionally analyzed within APOE4 carrier and non-carrier strata separately. MNA scores were mean-centered before interaction terms were formed, so that the APOE4 coefficient represents the adjusted difference between carriers and non-carriers at the mean MNA score; standardized coefficients (β) with 95% CIs are reported.

To examine which individual components of the MNA account for the cognitive association while avoiding the instability of selecting freely among all 18 items at this sample size, we adopted a domain-based strategy. Following the content structure of the MNA, the assessment-related items were grouped a priori into three conceptual nutritional domains: subjective wellbeing (self-perceived nutrition and health; items O and P), dietary content (meal frequency and protein, fruit/vegetable, and fluid intake; items J, K, L, and M), and anthropometric/muscle status (body mass index, mid-arm circumference, and calf circumference; items F, Q, and R) (Supplementary Table S1). Items reflecting acute or state-dependent change (A, B, D), functional or living circumstances (C, G, N), and clinical comorbidity (H, I) were not treated as candidate nutritional exposures; the neuropsychological-problems item (E) was likewise excluded a priori because it overlaps conceptually with the cognitive outcome. Within each domain, forward stepwise selection (entry/removal thresholds *p* = 0.05/0.10) was applied as an exploratory step to identify the single item most strongly associated with TS, adjusting for age, sex, education, APOE4, and VRS. The resulting domain-representative items were then entered simultaneously with full covariate adjustment in a domain-representative multivariable model, and variance inflation factors (VIF) were examined to confirm the absence of collinearity. These covariates were prespecified as established confounders of the nutrition–cognition association; variables that overlap with the cognitive outcome—most notably diagnostic group, which is defined in part by cognitive testing—were reserved for the domain-representative multivariable model with full (Model 2) covariate adjustment to avoid over-adjustment during item selection. The same three domain-representative items were identified under this covariate set. Because this item-level analysis is exploratory, the three domain-representative items were evaluated as a separate family of tests against a Bonferroni-corrected threshold of *p* < 0.0167 (0.05/3), and all item-level findings are interpreted as hypothesis-generating. Because item selection and the subsequent multivariable model were performed in the same sample, the reported *p* values do not account for the selection step, and the multivariable model should not be regarded as confirmatory. To assess reproducibility, the cohort was partitioned into two halves stratified by APOE4 status and clinical diagnosis, and the within-domain selection was repeated in each half; reselection of the same items across subsamples was regarded as evidence of internal reproducibility rather than as independent external validation (Supplementary Table S3). In addition, two sensitivity analyses evaluated the robustness of the subscale-level associations: one repeating all Model 2 analyses after removing clinical diagnosis from the covariate set (because diagnosis was based in part on cognitive testing and shares partial measurement overlap with TS), and one fitting a parsimonious core-adjusted model including only age, sex, education, APOE4, and VRS to address model over-specification given the sample size; both were performed for MNA-T, MNA-S, and MNA-A overall and within APOE4 strata (Supplementary Table S2). To examine whether the subscale–cognition associations were confounded by, or contingent upon, cognitive diagnostic status, the Model 1 and Model 2 analyses were additionally repeated separately within the cognitively normal and MCI strata (Supplementary Table S4). To confirm the robustness of our regression analyses, we assessed the assumptions of normality and homoscedasticity of residuals, and verified the absence of collinearity by utilizing normal probability plots, scatter plots, and variance inflation factor (VIF) values. All statistical analyses were performed using IBM SPSS Statistics, version 31.0 (IBM Corp., Armonk, NY, USA).

## 3. Results

### 3.1. Participant Characteristics

The demographic and clinical characteristics of the 196 participants, stratified by APOE4 status (156 APOE4 non-carriers, 40 APOE4 carriers), are summarized in Table 1. Mean age was 72.65 years (SD 5.95), 70.4% were female, and 57.7% met criteria for MCI. APOE4 non-carriers and carriers did not differ significantly in age, sex, education, MCI proportion, vascular risk, MMSE, physical activity, serum nutritional markers, MNA scores, or CERAD measures (all *p* > 0.05), although the modest APOE4 carrier subgroup size (*n* = 40) limits the power of these comparisons.

### 3.2. MNA Scores and Global Cognition: Subscale-Level Findings

In the overall sample, higher MNA-T was associated with higher TS in Model 1 (β = 0.374, 95% CI: 0.243 to 0.505, *p* < 0.001) and remained significant in the fully adjusted Model 2 (β = 0.151, 95% CI: 0.044 to 0.258, *p* = 0.006) (Table 2). MNA-S was significant in Model 1 (β = 0.247, 95% CI: 0.110 to 0.384, *p* < 0.001) but attenuated to marginal significance in Model 2 (β = 0.099, 95% CI: −0.006 to 0.204, *p* = 0.065). For MNA-A, β = 0.415, 95% CI: 0.286 to 0.544, *p* < 0.001 in Model 1, and β = 0.167, 95% CI: 0.059 to 0.275, *p* = 0.003 in Model 2. Of the three MNA scores, MNA-A showed the strongest adjusted association with TS, although the coefficients were not formally compared; the association for MNA-S was attenuated and did not reach statistical significance after adjustment.

### 3.3. APOE4 Moderation of the MNA–Cognition Relationship

**Figure 1:**
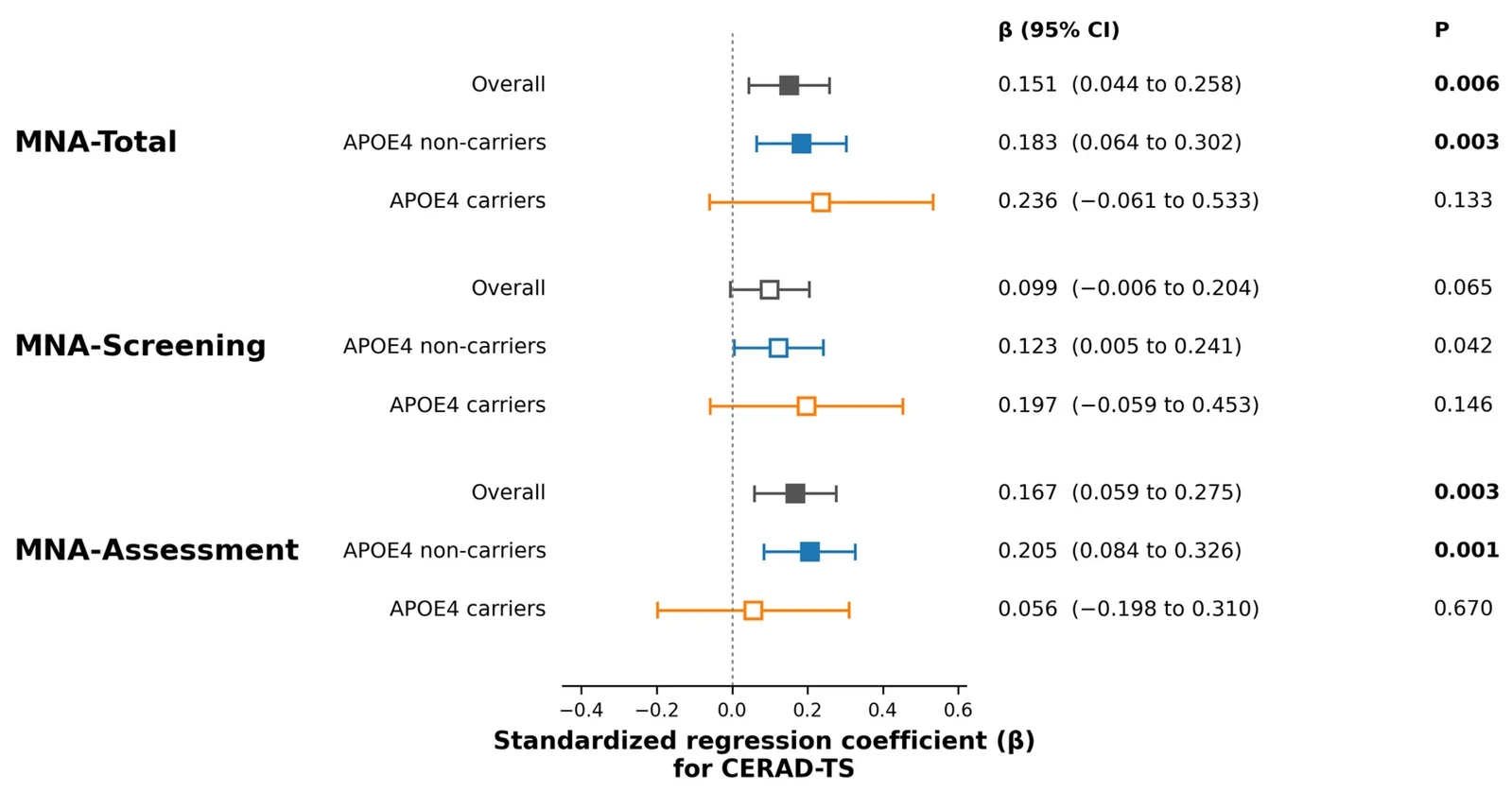
Standardized regression coefficients (β) of MNA scores for the prediction of CERAD total score (TS), overall and by APOE4 status, from the fully adjusted Model 2. Squares represent point estimates of β; horizontal bars indicate 95% confidence intervals (95% CI). Filled squares and bold *p*-values denote associations significant at the Bonferroni-corrected threshold (*p* < 0.0167 = 0.05/3); open squares and plain *p*-values denote non-significant associations. Grey, overall sample; blue, APOE4 non-carriers; orange, APOE4 carriers. Models were adjusted for all covariates. Significant associations were observed for MNA-Total and MNA-Assessment in the overall sample and in APOE4 non-carriers, but not in APOE4 carriers. The MNA-Screening subscale did not survive Bonferroni correction in any subgroup. The contrast was most pronounced for MNA-Assessment, for which the MNA-A × APOE4 interaction term was significant (*p* = 0.007): the standardized association was significant in APOE4 non-carriers (β = 0.205, 95% CI: 0.084 to 0.326, *p* = 0.001) but not in the smaller APOE4 carrier subgroup (β = 0.056, 95% CI: −0.198 to 0.310, *p* = 0.670), whose wide confidence interval reflects limited power in that stratum.

To formally test whether APOE4 status moderated the MNA–cognition relationship, multiple linear regression models incorporating two-way interaction terms (MNA score × APOE4 carrier status) were examined first (Table 3). The interaction terms were statistically significant for MNA-T (β = −0.163, 95% CI: −0.276 to −0.050, *p* = 0.005) and MNA-A (β = −0.162, 95% CI: −0.279 to −0.045, *p* = 0.007), indicating that the association between nutritional status and global cognition was significantly attenuated in APOE4 carriers. The MNA-S interaction term was in the same direction and approached, but did not reach, conventional significance (β = −0.110, 95% CI: −0.224 to 0.004, *p* = 0.059). In stratified analyses (Table 2; Figure 1), associations were evident in APOE4 non-carriers, whereas estimates in APOE4 carriers were imprecise. Among APOE4 non-carriers, MNA-T (β = 0.183, 95% CI: 0.064 to 0.302, *p* = 0.003) and MNA-A (β = 0.205, 95% CI: 0.084 to 0.326, *p* = 0.001) were associated with TS in the fully adjusted Model 2, while MNA-S reached only nominal significance (β = 0.123, 95% CI: 0.005 to 0.241, *p* = 0.042). In contrast, among APOE4 carriers, none of the three MNA scores were significantly associated with TS in Model 2 (MNA-T: *p* = 0.133; MNA-S: *p* = 0.146; MNA-A: *p* = 0.670). The pattern was consistent in Model 1, where MNA-T, MNA-S, and MNA-A were each highly significant in APOE4 non-carriers (all *p* < 0.001) but none reached significance in APOE4 carriers (all *p* > 0.10). Because the carrier subgroup comprised only 40 participants, the stratified analyses in this group were underpowered, and the absence of statistically significant associations should not be interpreted as evidence of no association.

### 3.4. Individual MNA Items and Global Cognition: Exploratory Item-Level Analysis

To identify which specific components of the MNA account for the cognitive association, we applied the domain-based strategy described in the Methods. Exploratory within-domain selection identified one representative item from each of the three a priori nutritional domains: self-rated health relative to peers (item P) from the subjective-wellbeing domain, protein intake (item K—composite indicator of dairy, legume/egg, and meat/fish/poultry consumption) from the dietary-content domain, and calf circumference (item R) from the anthropometric/muscle domain. In the domain-representative multivariable model with full covariate adjustment, self-rated health relative to peers (item P) was associated with TS at the Bonferroni-corrected threshold (β = 0.183, 95% CI 0.055 to 0.311, *p* = 0.006), whereas protein intake (item K; β = 0.146, 95% CI 0.019 to 0.273, *p* = 0.025) and calf circumference (item R; β = 0.123, 95% CI 0.004 to 0.242, *p* = 0.043) showed nominal associations that did not survive correction and should be regarded as exploratory. All VIFs were < 1.30, indicating no meaningful collinearity; the model explained 35.0% of the variance in TS (Table 4).

### 3.5. Sensitivity Analyses

Two sensitivity analyses tested the robustness of the subscale-level associations (Supplementary Table S2). First, after excluding clinical diagnosis from the covariate set—thereby removing the covariate that shares partial measurement overlap with TS—MNA-T (β = 0.229, 95% CI 0.093 to 0.365), MNA-S (β = 0.162, 95% CI 0.066 to 0.258), and MNA-A (β = 0.243, 95% CI 0.098 to 0.388) were each associated with TS (all *p* < 0.001), with estimates larger than those of the fully adjusted model. Second, in the parsimonious core-adjusted model (age, sex, education, APOE4 carrier status, and VRS), MNA-T (β = 0.278, 95% CI 0.112 to 0.444), MNA-S (β = 0.204, 95% CI 0.082 to 0.326), and MNA-A (β = 0.291, 95% CI 0.118 to 0.464) were likewise associated with TS (all *p* < 0.001). In both sensitivity models, the standardized coefficients for all three scores were smaller in APOE4 carriers than in non-carriers (diagnosis-excluded: MNA-T, 0.199 vs. 0.265; MNA-S, 0.089 vs. 0.185; MNA-A, 0.133 vs. 0.292; core-adjusted: 0.087 vs. 0.313, 0.103 vs. 0.232, and 0.022 vs. 0.337, respectively), with wide confidence intervals in carriers, none of which excluded zero. Reproducibility of the domain-representative item selection was assessed by repeating the within-domain selection in two halves of the cohort stratified by APOE4 status and clinical diagnosis (Supplementary Table S3); the representative items were reselected as follows: item P in both of the two halves, item K in both, and item R in both, supporting internal reproducibility given the reduced power of half-sized samples.

To assess whether the MNA–cognition association merely reflected the pooling of cognitively normal and MCI participants or was driven by cognitive impairment itself, the analyses were repeated separately within each diagnostic stratum (Supplementary Table S4). In the fully adjusted Model 2, the associations were preserved in both diagnostic strata, with comparable effect sizes. The MNA-A associations survived Bonferroni correction in cognitively normal (β = 0.282, 95% CI: 0.105 to 0.459, *p* = 0.002) and in MCI participants (β = 0.209, 95% CI: 0.043 to 0.375, *p* = 0.015), as did MNA-T in the MCI stratum (β = 0.216, 95% CI: 0.049 to 0.383, *p* = 0.011); MNA-T in cognitively normal participants (β = 0.199, 95% CI: 0.020 to 0.378, *p* = 0.029) and MNA-S in the MCI stratum (β = 0.175, 95% CI: 0.009 to 0.341, *p* = 0.040) reached nominal significance. Because the association was present in cognitively normal individuals—in whom reverse causation from established cognitive impairment is least plausible—and did not depend on the MCI subgroup, these results suggest that the MNA–cognition relationship is not solely a downstream consequence of established cognitive impairment, although subclinical prodromal processes cannot be excluded. Given the smaller stratum sizes (cognitively normal, *n* = 83; MCI, *n* = 113), these analyses are exploratory and were interpreted with the corresponding reduction in power.

## 4. Discussion

This study provides three main findings on the relationship between nutritional status and cognition in non-demented older adults. First, by decomposing the MNA into its screening and assessment subscales, we observed that the detailed assessment subscale (MNA-A) showed a stronger adjusted association with global cognition than the brief screening subscale (MNA-S), whose association did not reach statistical significance after multivariable adjustment. Second, by formally testing APOE4 as a moderator, we observed that the MNA–cognition association differed by APOE4 carrier status: significant associations were observed in APOE4 non-carriers but not in the smaller APOE4 carrier subgroup (*n* = 40), despite similar mean MNA scores between groups (Figure 1). Third, by grouping the 18 individual MNA items a priori into three conceptually distinct nutritional domains and identifying one representative item within each, we examined three items—self-rated health relative to peers (subjective wellbeing), protein intake (dietary content), and calf circumference (lower-limb muscle reserve)—of which only self-rated health remained significant after correction for multiple comparisons. Together, these hypothesis-generating findings suggest that the association between nutritional status and cognition differs by APOE4 carrier status and may involve several nutritional domains.

Our overall finding that better MNA status is associated with better global cognition is consistent with prior community-based and clinic-based studies. In the Korean Brain Aging Study for the Early Diagnosis and Prediction of Alzheimer’s Disease (KBASE), dementia was an independent predictor of malnutrition risk, and lower nutritional status was related to cortical thinning in the left temporal regions [10]. In the Singapore Longitudinal Ageing Studies, a global malnutrition risk score predicted incident cognitive decline and incident neurocognitive disorders over 3–5 years of follow-up, with low albumin, polypharmacy, low fruit/vegetable/milk intake, and low total cholesterol as the component drivers [9]. Cross-sectional analyses from the Longitudinal Aging Study in India–Diagnostic Assessment of Dementia (LASI-DAD) and from a large community-dwelling Lebanese cohort have likewise shown that MNA-defined malnutrition is strongly associated with cognitive impairment [11,12]. Our findings extend these reports in three specific ways. Because none of the biological pathways discussed below—such as neuroinflammation, sarcopenia-related processes, cerebral perfusion, and myokine signaling—were measured in this study, they should be regarded as possible explanations rather than tested mechanisms.

### 4.1. Subscale-Level Decomposition: A Stronger Association for the Assessment Subscale

Although the MNA screening subscale is often used in isolation for clinical convenience, our results indicate that, after adjustment for demographic, clinical, lifestyle, and metabolic covariates, the screening subscale alone is no longer significantly associated with global cognition (*p* = 0.065), whereas the assessment subscale remains highly significant (*p* = 0.003). Notably, the association for MNA-S was sensitive to adjustment for clinical diagnosis: it was significant when diagnosis was excluded from the covariate set and in the parsimonious model (both *p* < 0.001; Supplementary Table S2). This pattern may reflect content composition. The screening subscale is dominated by acute or state-dependent items (recent appetite loss, weight change, acute illness, mobility), which capture transient nutritional perturbations but may be only loosely coupled to the chronic processes that shape cognition. In contrast, the assessment subscale uniquely captures three slowly evolving constructs—dietary content (items K, L, M), self-perceived health (items O, P), and lower-limb anthropometry (item R)—which may relate to cognition through chronic inflammatory, metabolic, or sarcopenia-related pathways that act over years rather than days [3,4,31]. Thus, while the MNA-Short Form remains a useful triage tool, in our sample, the association with cognition was more evident for the detailed assessment subscale. Whether the full MNA, rather than the screening section alone, provides additional information about cognitive status, and whether it has any predictive value, requires prospective testing.

### 4.2. APOE4 Carrier Status and the Nutrition–Cognition Association

Several biological differences between APOE4 carriers and non-carriers could be relevant to the pattern observed here. APOE4 carriers exhibit greater amyloid-β accumulation, altered lipid handling, increased neuroinflammatory tone, and heightened oxidative stress, all of which converge on the same downstream pathways through which adequate nutrition is hypothesized to support cognition [15,16,17,18]. One possible explanation is that amyloidogenic and neuroinflammatory processes in APOE4 carriers attenuate the observable association between nutritional status and cognition; because amyloid, tau, and neuroinflammatory markers were not measured in this study, this explanation requires confirmation. Prior evidence on whether APOE4 carrier status modifies the cognitive associations of modifiable factors is mixed: some studies have reported stronger associations in carriers, for example for social engagement and mindfulness [32], whereas an umbrella review suggested that older carriers may be less likely to benefit from several dietary interventions [21]. For MNA-defined nutritional status, our results are more consistent with the latter pattern: the interaction terms with APOE4 carrier status were significant for MNA-T and MNA-A (Table 3), although the stratified estimates in carriers were imprecise.

This finding does not imply that nutrition is unimportant for APOE4 carriers—basic adequate nutrition remains essential for frailty prevention, sarcopenia mitigation, and general health. Rather, it raises the hypothesis-generating possibility that better nutritional status may be associated with a larger marginal cognitive difference in APOE4 non-carriers, even though APOE4 carriers carry higher absolute AD risk. Future interventional trials of nutritional or dietary-pattern interventions for cognitive preservation may therefore consider stratifying or randomizing by APOE4 carrier status, both to maximize statistical power and to characterize genotype-specific effect sizes.

### 4.3. Item-Level Analysis: Three Nutritional Domains

The exploratory domain-based analysis identified one representative item per domain; of these, only item P remained significant after Bonferroni correction. Item P (self-rated health relative to people of the same age) entered first and retained the largest standardized effect across all models. Self-rated health is a validated predictor of mortality and cognitive decline in older adults and is thought to integrate objective health status, subjective wellbeing, and early apathy or depressive symptoms that frequently coexist with malnutrition and MCI [33]. Its emergence as the strongest single MNA item suggests that subjective perception of one’s health captures cognitively relevant variance beyond what individual objective markers convey. That same breadth, however, means item P may partly index consequences of incipient cognitive or mood changes rather than nutrition alone; we therefore interpret it as a marker of overall nutritional–health status rather than a discrete, independently modifiable nutritional target.

Item K (protein intake—composite indicator of dairy, legume/egg, and meat/fish/poultry consumption) entered second and is consistent with growing evidence that adequate dietary protein supports muscle and brain health and that diets richer in protein sources are associated with slower cognitive decline [4,34]. Inadequate protein intake has been associated with sarcopenia, low-grade inflammation, and reduced neurotrophic factor production, which have in turn been linked to cognitive decline [35]. Because item K is a coarse, frequency-based indicator rather than a quantitative measure of protein intake (e.g., g·kg^−1^·day^−1^), its implications for protein as an intervention target should be confirmed with quantitative dietary assessment.

Item R (calf circumference, CC) was selected from the anthropometric domain. Calf circumference is a well-validated, non-invasive marker of lower-limb muscle mass and a screening tool for sarcopenia in older adults. Its nominal association with TS—beyond items P and K—is consistent with the hypothesis that sarcopenia is relevant to cognitive aging [36] and with prior work suggesting that lower-limb musculature may support cerebrovascular health, executive function, and attention through proposed mechanisms such as the muscle-pump effect on cerebral perfusion and myokine-driven neurotrophic signaling [37]. Notably, the BMI item (MNA-F) and the MAC item (MNA-Q) were not selected within the anthropometric domain in favor of calf circumference (item R), suggesting that CC captures cognitively relevant anthropometric variance that BMI and MAC do not—a finding that aligns with our group’s previous report from the same cohort showing that CC was specifically associated with non-memory cognitive domains [38].

Taken together, the three items span distinct nutritional domains—subjective wellbeing, dietary content, and anthropometric muscle reserve. Although only item P survived correction for multiple comparisons, the selection of one representative item from each domain is consistent with contributions from several nutritional domains, which should be confirmed in independent cohorts. Likewise, whether multidomain nutritional approaches improve cognition requires interventional testing.

### 4.4. Study Limitations and Future Directions

Several limitations should be acknowledged, in order of importance. First, the APOE4 carrier subgroup was small (*n* = 40), and all but one carrier were ε3/ε4 heterozygotes. Analyses in this group were underpowered, the absence of significant associations should not be interpreted as evidence of no association, and gene-dosage effects could not be assessed; replication in larger cohorts enriched for carriers is needed. Second, the cross-sectional design precludes causal inference. Reverse causation is plausible, because early or prodromal cognitive impairment can reduce appetite, food acquisition, and physical activity. Nevertheless, the associations were also present among cognitively normal participants (Supplementary Table S4), arguing against reverse causation as the sole explanation; future prospective studies are needed to establish the temporal direction of these associations. Third, generalizability is limited. Participants were drawn from a single Korean cohort recruited mainly through a memory-clinic dementia screening program, which may have selected individuals with greater cognitive concern or access to care, and the MNA food items, particularly item K, reflect a dietary culture that may differ elsewhere. Fourth, the domain-representative items were selected by forward stepwise selection, a data-dependent procedure that can yield unstable coefficients and inflate Type I error, in the same sample in which they were evaluated; the item-level findings are therefore exploratory and require validation in independent cohorts. Fifth, nutritional status was assessed only with the MNA, which does not capture detailed dietary patterns such as the Mediterranean, DASH, or MIND diets [4,39] and assesses protein intake only with item K, a coarse frequency-based indicator; quantitative dietary intake measures, inflammatory markers, and blood-based AD biomarkers (amyloid-β, p-tau) were also unavailable, so the proposed biological pathways could not be examined. In addition, several MNA items reflect general health, functional status, and psychological well-being rather than nutrition per se, and the MNA-T and MNA-S scores include item E (neuropsychological problems), which overlaps conceptually with cognition; the observed associations may therefore not be specific to nutrition. Finally, the fully adjusted models included many covariates relative to the sample size, particularly within the carrier stratum, and residual confounding by unmeasured or imprecisely measured factors cannot be excluded.

## 5. Conclusions

In this cross-sectional analysis of non-demented older Korean adults, better nutritional status as measured by the MNA, particularly its assessment subscale, was associated with better global cognition after adjustment for demographic, clinical, lifestyle, and metabolic covariates. This association differed by APOE4 carrier status and was evident predominantly in non-carriers, although the small carrier subgroup limits inference in carriers. In exploratory item-level analyses, self-rated health relative to peers was associated with global cognition after correction for multiple comparisons, whereas the associations of protein intake and calf circumference were nominal. Methodologically, the full 18-item MNA, rather than the screening section alone, may be preferable for the assessment of nutritional correlates of cognitive status. These findings are hypothesis-generating and should be tested in larger longitudinal and interventional studies incorporating relevant biological markers, with APOE4 carrier status prespecified as a stratification or interaction variable.

## Figures and Tables

**Table 1:** Clinical characteristics of the participants according to APOE4 status.

	Overall	APOE4 Non-Carriers	APOE4 Carriers	*p*
*n*	196	156	40	
Age, y	72.65 (5.95)	72.95 (5.96)	71.50 (5.86)	0.170 ^a^
Female, *n* (%)	138 (70.41)	106 (67.95)	32 (80.00)	0.136
Education, y	9.62 (4.51)	9.61 (4.55)	9.68 (4.38)	0.934 ^a^
MMSE	25.58 (3.45)	25.52 (3.46)	25.83 (3.43)	0.618 ^a^
MCI, *n* (%)	113 (57.65)	88 (56.41)	25 (62.50)	0.487
VRS, %	23.98 (18.58)	23.93 (19.14)	24.17 (16.43)	0.943 ^a^
PASE	64.77 (46.21)	64.45 (47.19)	66.04 (42.70)	0.847 ^a^
Annual income, *n* (%)				
<MCL	25 (12.76)	22 (14.10)	3 (7.50)	0.216 ^b^
MCL, <2MCL	62 (31.63)	52 (33.33)	10 (25.00)	
2MCL	109 (55.61)	82 (52.56)	27 (67.50)	
Alcohol intake status, *n* (%)				
Never/Former/Drinker	107 (54.59)/34 (17.35)/55 (28.06)	83 (53.21)/30 (19.23)/43 (27.56)	24 (60.00)/4 (10.00)/12 (30.00)	0.387 ^b^
Smoking status, *n* (%)				
Never/Former/Smoker	149 (76.02)/39 (19.90)/8 (4.08)	116 (74.36)/35 (22.44)/5 (3.21)	33 (82.50)/4 (10.00)/3 (7.50)	0.123 ^b^
Nutritional status				
MNA-T	25.60 (3.33)	25.46 (3.43)	26.15 (2.89)	0.247 ^a^
MNA-S	12.60 (2.08)	12.54 (2.13)	12.83 (1.91)	0.439 ^a^
MNA-A	13.01 (1.75)	12.93 (1.78)	13.33 (1.61)	0.200 ^a^
Serum nutritional markers				
Albumin, g/dL	4.57 (0.26)	4.57 (0.26)	4.60 (0.25)	0.465 ^a^
Glucose, fasting, mg/dL	108.15 (19.94)	108.46 (21.02)	106.87 (14.87)	0.660 ^a^
HDL-Cholesterol, mg/dL	54.64 (12.96)	54.51 (12.89)	55.21 (13.38)	0.765 ^a^
LDL-Cholesterol, mg/dL	96.41 (33.82)	96.10 (35.42)	97.68 (26.64)	0.796 ^a^
Cognition				
CERAD TS	69.98 (15.61)	70.00 (16.15)	69.90 (13.52)	0.971 ^a^

Abbreviations: APOE4, apolipoprotein E ε4 allele; MCI, mild cognitive impairment; VRS, vascular risk score; MMSE, mini-mental state examination; PASE, Physical Activity Scale for the Elderly; MNA-T, Mini-Nutritional Assessment total score; MNA-S, MNA screening subscore; MNA-A, MNA assessment subscore; HDL, high-density lipoprotein; LDL, low-density lipoprotein; CERAD, Consortium to Establish a Registry for Alzheimer’s Disease; TS, total score. Data are expressed as mean (standard deviation), unless otherwise indicated. ^a^ by one-way analysis of variance. ^b^ by chi-square test.

**Table 2:** Multiple linear regression of nutritional status and global cognition (CERAD total score), by APOE4 subgroup.

		Overall		APOE4 Non-Carriers		APOE4 Carriers	
	Model	β (95% CI)	*p*	β (95% CI)	*p*	β (95% CI)	*p*
**MNA-Total score**	Model 1	0.374 (0.243 to 0.505)	**<0.001**	0.401 (0.257 to 0.545)	**<0.001**	0.274 (−0.045 to 0.593)	0.101
	Model 2	0.151 (0.044 to 0.258)	**0.006**	0.183 (0.064 to 0.302)	**0.003**	0.236 (−0.061 to 0.533)	0.133
**MNA-Screening**	Model 1	0.247 (0.110 to 0.384)	**<0.001**	0.270 (0.118 to 0.422)	**<0.001**	0.226 (−0.097 to 0.549)	0.178
	Model 2	0.099 (−0.006 to 0.204)	0.065	0.123 (0.005 to 0.241)	0.042	0.197 (−0.059 to 0.453)	0.146
**MNA-Assessment**	Model 1	0.415 (0.286 to 0.544)	**<0.001**	0.446 (0.305 to 0.587)	**<0.001**	0.207 (−0.116 to 0.530)	0.218
	Model 2	0.167 (0.059 to 0.275)	**0.003**	0.205 (0.084 to 0.326)	**0.001**	0.056 (−0.198 to 0.310)	0.670

Abbreviations: MNA, Mini-Nutritional Assessment; APOE4, apolipoprotein E ε4 allele; VRS, vascular risk score. Model 1: no covariates. Model 2: adjusted for age, sex, APOE4 (overall analysis only), education, clinical diagnosis, VRS, PASE score, annual income, alcohol intake, smoking, albumin, fasting glucose, and HDL- and LDL-cholesterol. Bold *p* values indicate significance at the Bonferroni-corrected threshold (*p* < 0.0167) in both Model 1 and Model 2.

**Table 3:** Interaction of mean-centered nutritional status and APOE4 carrier status in predicting global cognition (CERAD total score).

	β (95% CI)	*p*
**MNA-Total score**		
MNA-T (centered)	0.207 (0.092 to 0.322)	**<0.001**
APOE4 carrier status	0.014 (−0.091 to 0.119)	0.792
MNA-T × APOE4 carrier status	−0.163 (−0.276 to −0.050)	**0.005**
**MNA-Screening**		
MNA-S (centered)	0.126 (0.015 to 0.237)	**0.026**
APOE4 carrier status	−0.016 (−0.124 to 0.092)	0.771
MNA-S × APOE4 carrier status	−0.110 (−0.224 to 0.004)	0.059
**MNA-Assessment**		
MNA-A (centered)	0.247 (0.124 to 0.370)	**<0.001**
APOE4 carrier status	−0.003 (−0.105 to 0.100)	0.958
MNA-A × APOE4 carrier status	−0.162 (−0.279 to −0.045)	**0.007**

Abbreviations: MNA-T, Mini-Nutritional Assessment total score; MNA-S, MNA screening subscore; MNA-A, MNA assessment subscore; APOE4, apolipoprotein E ε4 allele; TS, total score of the Consortium to Establish a Registry for Alzheimer’s Disease; β, standardized regression coefficient; CI, confidence interval. To explore the moderating effects of APOE4 carrier status on the associations between nutritional status and global cognition (TS), multiple linear regression analyses were performed including two-way interaction terms between nutritional status and APOE4 carrier status as additional independent variables, adjusting for all Model 2 covariates. MNA scores were mean-centered before interaction terms were formed, and APOE4 carrier status was coded 0 for non-carriers and 1 for carriers; the APOE4 coefficient therefore represents the adjusted difference in TS between carriers and non-carriers at the mean MNA score. Bold *p* values indicate *p* < 0.05.

**Table 4:** Domain-representative multivariable model of MNA items predicting global cognition (CERAD total score).

MNA Item	Domain	β (95% CI)	B (95% CI)	VIF	*p*
Self-rated health vs. peers (item P)	Subjective wellbeing	0.183 (0.055 to 0.311)	5.880 (1.733 to 10.027)	1.286	0.006
Protein intake (item K)	Dietary content	0.146 (0.019 to 0.273)	6.342 (0.795 to 11.890)	1.249	0.025
Calf circumference (item R)	Anthropometry/muscle	0.123 (0.004 to 0.242)	5.730 (0.171 to 11.289)	1.094	0.043

One representative item per a priori nutritional domain (Supplementary Table S1), identified by exploratory within-domain forward selection, was entered simultaneously with full covariate adjustment (age, sex, APOE4, education, clinical diagnosis, VRS, PASE score, income, alcohol, smoking, albumin, fasting glucose, and HDL- and LDL-cholesterol). β, standardized coefficient; B, unstandardized coefficient; VIF, variance inflation factor. Bonferroni-corrected threshold for the three domain-representative items, *p* < 0.0167; only item P met this threshold. Adjusted R^2^ = 0.350, *p* < 0.001.

## Data Availability

Data are not freely accessible because the IRB of Hallym University Dongtan Sacred Heart Hospital prevents public sharing for privacy reasons. Data are available on reasonable request after IRB approval (contact: yoon4645@gmail.com).

## Supplementary Materials

The following supporting information can be downloaded at https://www.mdpi.com/article/10.3390/nu18193217/s1: Table S1: A priori classification of MNA items into nutritional domains; Table S2: Sensitivity analyses of MNA subscores and global cognition; Table S3: Reproducibility of domain-representative item selection in stratified split-half analysis; Table S4: Multiple linear regression of nutritional status and cognition, by clinical diagnosis subgroup.

## Abbreviations

The following abbreviations are used in this manuscript:

AD	Alzheimer’s disease
APOE4	Apolipoprotein E ε4 allele
BMI	Body mass index
CC	Calf circumference
CDR	Clinical Dementia Rating
CERAD	Consortium to Establish a Registry for Alzheimer’s Disease
CI	Confidence interval
CN	Cognitively normal
HDL	High-density lipoprotein
LDL	Low-density lipoprotein
MAC	Mid-arm circumference
MCI	Mild cognitive impairment
MMSE	Mini-Mental State Examination
MNA	Mini-Nutritional Assessment
MNA-A	MNA assessment subscore
MNA-S	MNA screening subscore
MNA-T	MNA total score
PASE	Physical Activity Scale for the Elderly
TS	Total score
VIF	Variance inflation factor
VRS	Vascular risk score
