# Supplementary material for: Nutritional Status and Cognition in Older Adults: Modification by Apolipoprotein E ε4
Source: Nutrients. 2026 Sep 29;18(19):3217. doi: 10.3390/nu18193217 (PMC13667144; doi:10.3390/nu18193217)
Supplement: Supplementary file 1 [file nutrients-18-03217-s001.zip › nutrients-4556188-supplementary.pdf]

Table S1. A priori classification of MNA items into nutritional domains

Nutritional domain	MNA items (label)	Representative item
Subjective wellbeing	O (self-perceived nutritional status), P (self-rated health relative to peers)	P
Dietary content	J (number of full meals), K (protein intake), L (fruit/vegetable intake), M (fluid intake)	K
Anthropometry/muscle	F (body mass index), Q (mid-arm circumference), R (calf circumference)	R
Not treated as nutritional exposures	A, B, D (acute/state-dependent change); C, G, N (functional/living circumstances); H, I (clinical comorbidity); E (neuropsychological problems, overlapping with the cognitive outcome)	—

Items were grouped a priori following the content structure of the MNA. Within each nutritional domain, the representative item was identified by exploratory forward selection adjusting for age, sex, education, APOE4, and VRS.

Table S2. Sensitivity analyses of MNA subscores and global cognition

Model	Overall β (95% CI)	P	APOE4 non-carriers β (95% CI)	P	APOE4 carriers β (95% CI)	P
**MNA-Total score**						
Diagnosis-excluded	0.229 (0.093 to 0.365)	<0.001	0.265 (0.107 to 0.423)	<0.001	0.199 (−0.175 to 0.573)	0.297
Core-adjusted	0.278 (0.112 to 0.444)	<0.001	0.313 (0.127 to 0.499)	<0.001	0.087 (−0.256 to 0.430)	0.619
**MNA-Screening**						
Diagnosis-excluded	0.162 (0.066 to 0.258)	<0.001	0.185 (0.051 to 0.319)	0.007	0.089 (−0.311 to 0.489)	0.663
Core-adjusted	0.204 (0.082 to 0.326)	<0.001	0.232 (0.094 to 0.370)	<0.001	0.103 (−0.227 to 0.433)	0.541
**MNA-Assessment**						
Diagnosis-excluded	0.243 (0.098 to 0.388)	<0.001	0.292 (0.118 to 0.466)	<0.001	0.133 (−0.132 to 0.398)	0.326
Core-adjusted	0.291 (0.118 to 0.464)	<0.001	0.337 (0.136 to 0.538)	<0.001	0.022 (−0.325 to 0.369)	0.901

Multiple linear regression with CERAD total score as the dependent variable. Diagnosis-excluded: full covariate set (as in Table 2, Model 2) with clinical diagnosis (CN vs. MCI) removed. Core-adjusted: age, sex, education, APOE4, and VRS. APOE4 carrier and non-carrier strata exclude the APOE4 term. β, standardized coefficient.

Table S3. Reproducibility of domain-representative item selection in stratified split-half analysis

Nutritional domain	Representative item (full sample)	Selected in Half A	Selected in Half B
Subjective wellbeing	Self-rated health vs. peers (item P)	*Yes*	*Yes*
Dietary content	Protein intake (item K)	*Yes*	*Yes*
Anthropometry/muscle	Calf circumference (item R)	*Yes*	*Yes*

The cohort was partitioned into two halves stratified by APOE4 status and clinical diagnosis; within-domain forward selection was repeated in each half. Entries indicate whether the full-sample representative item was reselected (Yes/No). n (Half A) = 98; n (Half B) = 98.

Table S4. Multiple linear regression of nutritional status and cognition, by clinical diagnosis subgroup

	Overall		CN		MCI	
	**β (95% CI)**	**P**	**β (95% CI)**	**P**	**β (95% CI)**	**P**
MNA-Total score						
Model 1	0.374 (0.243 to 0.505)	**<0.001**	0.365 (0.185 to 0.545)	**<0.001**	0.263 (0.080 to 0.446)	**0.005**
Model 2	0.151 (0.044 to 0.258)	**0.006**	0.199 (0.020 to 0.378)	0.029	0.216 (0.049 to 0.383)	**0.011**
MNA-Screening						
Model 1	0.247 (0.110 to 0.384)	**<0.001**	0.245 (0.061 to 0.429)	**0.009**	0.167 (-0.018 to 0.352)	0.078
Model 2	0.099 (-0.006 to 0.204)	0.065	0.143 (-0.016 to 0.302)	0.076	0.175 (0.009 to 0.341)	0.040
MNA-Assessment						
Model 1	0.415 (0.286 to 0.544)	**<0.001**	0.425 (0.243 to 0.607)	**< 0.001**	0.312 (0.129 to 0.495)	**< 0.001**
Model 2	0.167 (0.059 to 0.275)	**0.003**	0.282 (0.105 to 0.459)	**0.002**	0.209 (0.043 to 0.375)	**0.015**

Abbreviations: MNA, Mini-Nutritional Assessment; CN, cognitively normal; MCI, mild cognitive impairment; APOE4, apolipoprotein E ε4 allele; VRS, vascular risk score.
Dependent variable: CERAD total score (TS). Model 1: no covariates. Model 2: adjusted for age, sex, APOE4, education, VRS, PASE score, annual income, alcohol intake, smoking, albumin, fasting glucose, and HDL- and LDL-cholesterol. Bold P values indicate significance at the Bonferroni-corrected threshold (P_B < 0.0167) in both Model 1 and Model 2.
